# Multi-equilibrium property of metabolic networks: SSI module

**DOI:** 10.1186/1752-0509-5-S1-S15

**Published:** 2011-06-20

**Authors:** Hong-Bo Lei, Ji-Feng Zhang, Luonan Chen

**Affiliations:** 1 Key Laboratory of Systems and Control, Academy of Mathematics and Systems Science, Chinese Academy of Sciences, Beijing 100190, China; 2 Key Laboratory of Systems Biology, Shanghai Institutes for Biological Sciences, Chinese Academy of Sciences, Shanghai 200031, China; 3 Collaborative Research Center for Innovative Mathematical Modelling, Institute of Industrial Science, University of Tokyo, Tokyo 153-8505, Japan

## Abstract

**Background:**

Revealing the multi-equilibrium property of a metabolic network is a fundamental and important topic in systems biology. Due to the complexity of the metabolic network, it is generally a difficult task to study the problem as a whole from both analytical and numerical viewpoint. On the other hand, the structure-oriented modularization idea is a good choice to overcome such a difficulty, i.e. decomposing the network into several basic building blocks and then studying the whole network through investigating the dynamical characteristics of the basic building blocks and their interactions. Single substrate and single product with inhibition (SSI) metabolic module is one type of the basic building blocks of metabolic networks, and its multi-equilibrium property has important influence on that of the whole metabolic networks.

**Results:**

In this paper, we describe what the SSI metabolic module is, characterize the rates of the metabolic reactions by Hill kinetics and give a unified model for SSI modules by using a set of nonlinear ordinary differential equations with multi-variables. Specifically, a sufficient and necessary condition is first given to describe the injectivity of a class of nonlinear systems, and then, the sufficient condition is used to study the multi-equilibrium property of SSI modules. As a main theoretical result, for the SSI modules in which each reaction has no more than one inhibitor, a sufficient condition is derived to rule out multiple equilibria, i.e. the Jacobian matrix of its rate function is nonsingular everywhere.

**Conclusions:**

In summary, we describe SSI modules and give a general modeling framework based on Hill kinetics, and provide a sufficient condition for ruling out multiple equilibria of a key type of SSI module.

## Background

Revealing the multi-equilibrium property of a metabolic network is a fundamental and important topic in systems biology [1-5]. Generally, it is not only expensive but also difficult, if not impossible, to solve this problem via biological experiments. Hence, a systematical modeling approach is strongly demanded [6-8]. However, in the traditional theoretical analysis, necessary information on model parameters is always required. Due to the limitation of measurement tools, measurement errors and biological variability, most of the model parameters are either unavailable or uncertain. This not only makes it difficult to analyze the model, but also limits the applications of the theoretical results based on a model with fixed parameter values. In contrast to detailed model parameters, the topological structure of a metabolic network is relatively easier to be obtained and is invariant for many cases. Hence, a structure-oriented analysis should be much more useful on understanding qualitative dynamics of metabolic networks, since it can not only overcome the difficulty due to the lack of parameter information, but also provide a deep insight into the essential design principles.

There are some pioneering works in structure-oriented study on multiple equilibria of networks [3,9-17], which have recently been surveyed in [5]. A metabolic network in a living cell is a large-scale molecular network and contains a great number of metabolites and reactions, and thus, is generally difficult to be theoretically analyzed as a whole, especially when there is no parameters but only structure information available.

To overcome such a difficulty, we proposed a structure-oriented modularization framework in [5]: using the modularization idea commonly used in the area of control theory [18,19], viewing a metabolic network as an assembly of basic building blocks (called metabolic modules) with specific structures, and investigating the multi-equilibrium property of the original network by studying the characteristics of these basic modules and their interactions. Such an idea not only reduces the difficulty in investigating a complex metabolic network, but also makes full use of the structure information, thereby overcomes the limitation of the methods based on models with fixed parameter values. After getting a deep insight of the basic building blocks, people can use them to reconstruct new metabolic networks.

In particular, in [5] we showed that a metabolic network can be decomposed into four types of basic modules according to the topological structure, and proved that one type of those modules, i.e. the single substrate and single product with no inhibition (SSN) modules, cannot admit multiple equilibria. Here we will focus on another important type of those basic modules, i.e. the single substrate and single product with inhibition (SSI) modules, and investigate their multi-equilibrium property.

Comparing with SSN modules, an SSI module contains metabolic reactions which are inhibited by other metabolites. Hence, the topological structure of an SSI module is much more complex from theoretical viewpoint. The metabolites interconnect with each other via reactions without inhibitions in SSN modules, while via reactions with inhibitions in SSI modules. Inhibitions make the metabolites (state variables) couple with each other in SSI modules, which are actually a kind of negative feedbacks. Moreover, the reaction mechanisms are much more complicated in SSI modules than those in SSN modules. For instance, when the other conditions (such as temperature, pH, the concentration and activity of the enzymes) are unchanged, the reaction rates depend mainly on the substrate concentrations in SSN modules but are simultaneously affected by the substrates, the inhibitions and their interactions in SSI modules.

Owing to these inherent characteristics, both the modeling procedure and theoretical analysis for SSI modules are much more difficult than those for SSN modules. Specifically, first, the intricate topological structure makes the modeling procedure for SSI modules much complicated. It is relatively easy to describe the rate of a metabolic reaction based on Hill kinetics if its inhibitors are known. But in a general SSI module, each reaction may be inhibited by other metabolites, and each metabolite may act as an inhibitor for other reactions. Hence, it is difficult to construct a unified model for SSI modules. Second, the strong coupling in SSI modules makes the model difficult to analysis. The metabolites mutually restrain each other via inhibitions in SSI modules, which may result in a loop or other complex structure.

Therefore, we have to consider all the metabolites simultaneously, which makes the dimension reduction of the system useless. Third, the complicated mechanisms of the reactions in SSI modules make the reaction rate equations more complex. In fact, the reaction rate is an increasing function of one variable in SSN modules, and is a polynomial that is increasing in any of its variables in the work [3,16,17] of Craciun et al.. But, in SSI modules, the reaction rate involves two or more variables, and is increasing in the concentration of substrate and decreasing in the concentration of inhibitor, which is also the essential difference between this work and that of Craciun et al..

The above characteristics of SSI modules makes the analytical skills developed for the SSN module cases no longer applicable. To overcome these difficulties, we first construct a special vector space, and represent the unified model of SSI modules via a system of nonlinear ordinary differential equations in a vector form. And then, we investigate the multi-equilibrium property of SSI modules through analyzing a sufficient and necessary condition of the injectivity of a particular nonlinear system. For the SSI modules in which each reaction has at most one inhibitor, we derive a sufficient condition for the absence of multiple equilibria, i.e. the Jacobian matrix of the rate function is nonsingular everywhere.

## Results and Discussion

### SSI metabolic module

If a metabolite can bind the enzyme of a metabolic reaction to repress its activity and decrease the reaction rate, then it is generally called an inhibitor of the enzyme or the reaction. This process is called the inhibition of the enzyme or the reaction. Generally, it is difficult to investigate a reaction with inhibition from the viewpoints of both experiment and theory, and special analysis methods is required. Hence, to investigate a metabolic network, it may be necessary and feasible to divide the metabolic reactions into two groups, one is with inhibition and the other is with no inhibition. In real metabolic networks, many reactions are with only one substrate and one product. Compared with other type of reactions, such reactions have particular properties, and is worth investigating first. Hence, we classified the metabolic reactions into four classes according to the number of substrates and products and the existence of inhibition [5].

**Definition 1.***(*[*5*]*) A metabolic reaction is called a single substrate and single product (SS) reaction, if it contains only one substrate and one product; otherwise, called a multiple substrates or multiple products (MM) reaction. An SS (or MM) reaction is called an SS (or MM) reaction with inhibition, SSI (or MMI) for short, if there exist some inhibitors of the reaction; otherwise, called an SS (or MM) reaction with no inhibition, SSN (or MMN) for short.*

**Remark 1.***A reversible reaction will be viewed as two reactions. For example, take**as the forward reaction**and the reverse reaction* .

Before giving the definition of the SSI module, we need the following concepts.

**Definition 2.***For a group of SS metabolic reactions ( including SSN and SSI reactions), take each metabolite as a node. If two nodes appear in a same reaction, link them with a directed edge (arrow) from the substrate to the product, and such an edge is called reaction edge. If a metabolite can inhibit some reaction, link it and the reaction edge with a line that contains a bar at the end near the reaction edge, and such an edge is called inhibition edge. Then we get a graph, called reaction graph of the group of SS reactions.*

**Figure 1:**
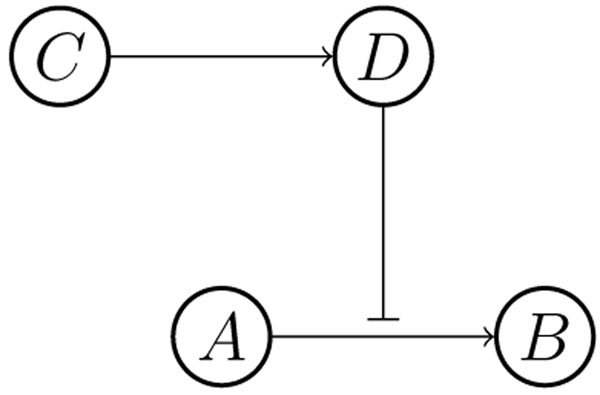
**A reaction graph** Each node means a metabolite. An arrow represents a reaction, and the bar at the end of a line denotes an inhibitor.

Now, we give an example to show how to get a reaction graph. Suppose that there are two SS reactions: *A* → *B*, *C* → *D*, and the metabolite *D* is an inhibitor of the first reaction. The corresponding reaction graph is shown in Figure 1.

**Definition 3.***In the reaction graph of a group of SS metabolic reactions, a node is called an input node, if the direction of each reaction edge connecting it points to other node; a node is called an output node, if the direction of each reaction edge connecting it points to itself. The other nodes are called state nodes. A state node that directly connects with an input (or output) node is called a head (or an end) node.*

**Definition 4.***(*[*5*]*) A reaction is said to be relevant to a metabolite S, if S is a reactant, a product or an inhibitor of this reaction*.

**Definition 5.***A path is a sequence of nodes such that from each of its nodes there is a directed reaction edge to the next node in the sequence*.

Now we can define the SSI module.

**Definition 6** (SSI module). *For a given metabolic network, denote  the set of all the metabolites, and  the set of all the reactions. The triple (ℒ, ℛ, ℐ) is called an SSI module within the metabolic network, ℒ, ℛ and ℐ are called the state node set, the reaction set and the inhibition set of the SSI module, respectively, if the following conditions are satisfied:*

(i)  is nonempty.

*(ii)  is nonempty and constituted of all the reactions which are relevant to the metabolites in* ℒ.

(iii) The reactions in ℛ are all SS (including SSN and SSI) reactions.

(*iv*) *ℐ* ⊂ ℒ × *ℛis nonempty*, *and its element* (*I*, *A* → *B*) *means the metabolite I is an inhibitor of the reaction A* → *B*.

(v) If there exist both input and output nodes, then for any S ∈ ℒ, there exist a directed path from some input node to some output node passing S in the reaction graph of ℛ.

(vi) The undirected graph constructed as follows is connected: remove all the input and output nodes, the inhibition edges, and the reaction edges connected with the input or output nodes in the reaction graph of ℛ; replace each directed reaction edge by an undirected one.

**Remark 2.***Not all reactions in an SSI module are SSI reactions.*

**Figure 2:**
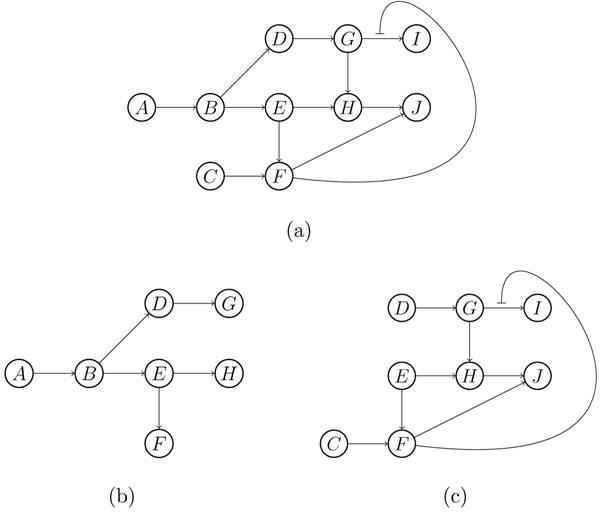
**An SSI module** The SSI module (a) can be decomposed into the SSN module (b) and the smaller SSI module (c).

**Figure 3:**
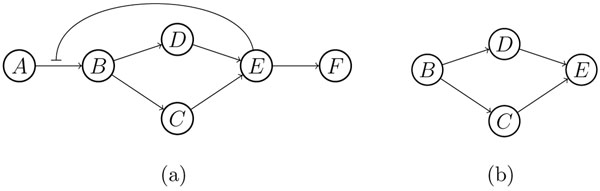
**An SSI module** The SSI module (a) cannot be decomposed into an SSN module and a new SSI module.

**Remark 3.***Although each reaction in an SSI module has single substrate and single product, an SSI module could be with multiple inputs and multiple outputs, i.e. having multiple input and output nodes. Furthermore, an SSI module could contain an SSN module or be decomposed into an SSN module and a smaller SSI module. For example, the SSI module shown in* Figure 2(a)*can be decomposed into the SSN module shown in*Figure 2(b)*and the SSI module shown in*Figure 2(c)*. But not all SSI modules can be decomposed in this way. For example, the SSI module of*Figure 3(a)* contains the SSN module shown*Figure 3(b)*, but cannot be decomposed any more. This question comes into the modularization decomposition of a metabolic network, which is beyond the scope of this paper.*

### Modeling SSI metabolic modules

We will give an appropriate expression to describe the rate of each metabolic reaction in an SSI module before modeling it in a mathematical manner, especially for the reactions with inhibition.

Two broad classes of enzyme inhibitions, i.e. irreversible and reversible, are generally recognized [20-23]. In an irreversible inhibition, the inhibitor combines with or destroys a functional group on the enzyme that is essential for its activity. The irreversible inhibitor dissociates very slowly from its target enzyme because it tightly binds to its active site. Such a process is always irreversible, and we do not consider it here. In contrast, in a reversible inhibition, the inhibitor dissociates very rapidly from its target enzyme because it becomes very loosely bound with the enzyme. Three types of reversible inhibitions are observed: competitive, uncompetitive and noncompetitive. Next we will introduce those reversible inhibitions [20-26]. A competitive inhibitor can combine reversibly with the active site of the enzyme and compete with the substrate. If the active site is occupied by the inhibitor, then it is unavailable for the binding of the substrate, which decreases the reaction rate. In the following reactions, the metabolite *I* is acting as a competitive inhibitor of the reaction *S* → *P*,

*S* + *E* ⇌ *SE* → *P* + *E*

*I* + *E* ⇌ *EI*,

where *S*, *E*, *P* and *I* are substrate, enzyme, product and inhibitor, respectively. Based on the Michaelis-Menten kinetics with the conservation condition on *E*, the rate of the reaction *S* → *P* can be described as(1)

where *C_S_* and *C_I_* represent the concentrations of the substrate *S* and the inhibitor *I*, respectively; *V_max_*means the maximum rate of the reaction, *K_M_* is the Michaelis-Menten constant, and *K_C_* is the competitive inhibition constant with respect to *I*.

An uncompetitive inhibitor cannot combine with a free enzyme, but only with an enzyme-substrate complex, and precludes the complex from converting into product. In the following reactions, the metabolite *I* is acting as a uncompetitive inhibitor of the reaction *S* → *P*,

*S* + *E* ⇌ *SE* → *P* + *E*

*I* + *SE* ⇌ *SEI*.

In this case, the rate of the reaction *S* → *P* can be described as(2)

where *K_U_* is the uncompetitive inhibition constant with respect to *I*.

An noncompetitive inhibitor can combine with both free enzyme and enzyme-substrate complexes. Enzyme is inactivated when such an inhibitor is bound, and cannot catalyze the conversion from substrate into product. In the following reactions, the metabolite *I* is acting as a noncompetitive inhibitor of the reaction *S* → *P*,

*S* + *E* ⇌ *SE* → *P* + *E*

*I* + *E* ⇌ *EI*

*I* + *SE* ⇌ *SEI*.

In this case, the rate of the reaction *S* → *P* can be described as(3)

Although the above three types of reversible inhibitions were observed in experiments, from the theoretical viewpoint, (3) is a general expression of (1) and (2) with appropriate parameter values. Hence, we will take (3) to describe the rate of reaction *S* → *P* when *I* is known to be an inhibitor.

Let (*ℒ*, *ℛ*, *ℐ*) be an SSI metabolic module containing *n* state nodes and *m* reactions, and denote

*ℒ* = {*S*_1_,⋯, *S_n_*},

*ℛ* = {*A*_1_ → *B*_1_,⋯, *A_m_* → *B_m_*}.

Assume that all the reactions in *ℛ* obey the Hill kinetics or the Michaelis-Menten kinetics. Then, for the reaction *A_j_* → *B_j_*, if *I_j_* is an inhibitor, then we use(4)

to describe the reaction rate; and if there is no inhibitor, then we take(5)

where *C_A_j__* and *C_I_j__* are the concentration of the metabolite *A_j_* and the inhibitor *I_j_*, *V_maxj_* represents the maximum rate of the reaction, *K_M_j__* is the Michaelis-Menten constant of the substrate *A_j_*, *n_j_* is the Hill coefficient, *K_j_* = (*K_M_j__*)*^n_j_^*. If *n_j_* = 1, (4) and (5) are also called Michaelis-Menten equations.

Let *C_i_* ≜ *C_S_i__* represent the concentration of the metabolite *S_i_*, and *C* = (*C*_1_,⋯, *C_n_*)*^τ^*, where *τ* means the transpose of a matrix. Note that the rate of change of the concentration of *S_i_* is given by the difference between the rate(s) of the reaction(s) generating *S_i_* and the rate(s) of the reaction(s) consuming *S_i_*. Then(6)

where *v_j_* is given by (4) if there exists inhibitor of the reaction and by (5) if there is no inhibitor. Then, we can get a model of the SSI module (*ℒ*, *ℛ*, *ℐ*),(7)

where *R_i_*(*C*; *P*) is given by the right hand side of (6), *P* is vector-valued model parameter. *R*(*C*; *P*) is called the rate function of the model.

**Remark 4.***If node A_j_ is an input node, then its concentration C_A_j__ in* (*4*) *or* (*5*) i*s not a variable of the model* (*7*) *but a parameter*.

**Definition 7.***For a fixed parameter P*_0_*, an equilibrium of ( 7 ) is a state C that satisfies  , i.e. a solution of the algebraic equations R(C; P*_0_*) = 0. System (7) or the SSI module (ℒ, ℛ, ℐ) is said to have the capability of multiple equilibria, if there exists a parameter P*_0_*such that the algebraic equations R(C;P*_0_*) = 0 have more than one positive solutions.*

### Theoretical results

In this section, we will derive a sufficient condition for the absence of multiple equilibria of a common type of SSI modules. But the system (7) is not effective for analyzing. So we convert it to another equivalent form first.

Define

We can show that ℝ*^ℒ^* is a vector space spanned by *ℒ* = {*S*_1_,⋯, *S_n_*}. For any reaction *A*→*B* ∈ *ℛ*, if *A* (or *B*) is a state node in *ℒ*, we view it as a vector in ℝ*^ℒ^* ; if *A* (or *B*) is an input (or output) node, we make a convention viewing it as the zero vector in ℝ*^ℒ^* . Denote

*ε_i_* = (0,⋯, 1,⋯, 0)*^τ^*,

whose entries are all zero except the *i*th position. Then

where

and the column vectors  and  in ℝ*^n^* are the coordinates of the vectors *B_j_* and *A_j_* in ℝ*^ℒ^* with respect to the basis *ℒ*, respectively. Thus, we get the equivalent model,(8)

Now, we can go on the model analysis based on the new equivalent model (8).

**Definition 8.***Mapping F*(*x*) : ℝ*^n^* → ℝ*^n^is called injective*, *if there does not exist x*_1_ ≠ *x*_2_ ∈ ℝ*^n^such that F*(*x*_1_) = *F*(*x*_2_).

**Lemma 1.***(*[*5*]*) Let F* : ℝ*^n^* → ℝ*^n^ be a map*, *and**** D**** be a subset of* ℝ*^n^*. *For a system of ordinary differential equations*

*if F (also called the vector field of the system) is injective in **D**, then the system cannot admit multiple equilibria in ** D**, i.e. the equations F*(*x*) = 0 *have no more than one root in ** D**.*

Lemma 1 provides a sufficient condition for the absence of multiple equilibria of a general system, but such a condition is difficult to be verified. Hence, we need to convert it into an equivalent one which is relatively easy to be verified. For some simple cases, for example, *f*(*x*) : ℝ → ℝ is continuously differentiable function of one variable, its injectivity is equivalent to that its differential is nonzero everywhere. Unfortunately, there is no such an equivalence for a general high dimensional map. As an counterexample, taking  , it is obvious that *F*(*x*, *y*) is injective on ℝ^2^, but the determinant of its Jacobian matrix is *det*(*JF*) = (*x* – 1)^2^, which is zero on line *x* = 1; and taking [27], the determinant of its Jacobian matrix is *det*(*JF*) ≡ 1, but F(0, *y* + 2*kπ*) = F (0, *y*), which means that *F* is not injective. Nevertheless, for some particular high dimensional map, its injectivity and the nonsingularity of its Jacobian matrix is equivalent. We will give such a class of maps in the following lemma.

**Lemma 2.***Suppose that l, n and m are some fixed positive integers. Let**** D*** ⊂ ℝ*^n^ be an open set,****P*** ⊂ ℝ*^l^, and ℛ =* {(*α_j_,β_j_*) : *α_j_,β_j_* ∈ ℝ*^n^,j =* 1*,*⋯*, m*}*. For a fixed parameter p* ∈ ***P****, let F*(·*,p*) : ℝ*^n^ →* ℝ*^n^ be a map of the following form,*(9)

*where x* = (*x*_1_,⋯, *x_n_*)*^τ^* ∈ ℝ^n^, *the function f_k_*(*·*,*p*) : ℝ^n^ → ℝ (*k* = 1,⋯, *m*) *is continuously differentiable with respect to x_i_* (*i* = 1,⋯, *n*).*Then*

*(i) if for any**and* , *there exist* , *and a nonzero vector**such that the following equation holds for any k* = 1,⋯,*m*,(10)

*then the condition that Jacobian matrix of F is nonsingular everywhere on**** D**** for any p* ∈ ***P****is sufficient to ensure that F is injective on**** D****for any p* ∈ ***P****;*

*(ii) if for any* , *and nonzero vector* , *there exist**and**such that (10) holds for all k* = 1, ⋯ ,*m*, *then the sufficient condition in* (*i*) *is also necessary*.

**Lemma 3.***Assume that n*, *m*, ℝ*^n^*, *** D****and ℛ have the same meanings as in Lemma 2*, *and* {*N*_1_, *N*_2_, *N*_3_, *N*_4_} *is a partition of N* = {1, ⋯ , *m*}, *i*.*e*. *they are disjoint and* . *Let* {*r_k_* : *k* ∈ *N*_3_ ∪ *N*_4_, *r_k_* ∈ {1, ⋯ , *n*}} *and* {*q_k_* : *k* ∈ *N*_2_ ∪ *N*_4_, *q_k_* ∈ {1, ⋯ , *n*}} *be two sequences and r_k_* ≠ *q_k_* .

Denote(11)(12)

*where a_k_*, *b_k_*, *c_k_*, *d_k_*, *u_k_* and *n_k_* ≥ 1 *are positive real number,*

*p* = (*p*_1_,⋯,*p_m_*) ∈ *** P****(**** P**** is corresponding parameter space)*.*Then the condition that Jacobian matrix of F is nonsingular everywhere on**** D**** for any p* ∈ ***P**** is equivalent to that F is injective on**** D**** for any p* ∈ *** P***.

**Thorem 1.***Let* (*ℒ*, *ℛ*, *ℐ*) *be an SSI module*, *and R*(*C;P*) *be the rate function of the corresponding model* (*8*). *Suppose that each reaction in ℛ has no more than one inhibitor*. *If the Jacobian matrix**is nonsingular for any P and C*, *then the model cannot admit multiple equilibria*.

### Discussion

**Figure 4:**
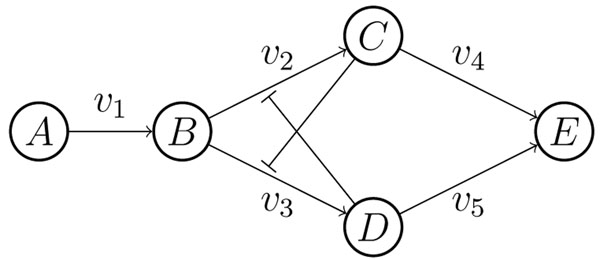
**An SSI module having multiple equilibria** This SSI module can admit multiple equilibria.

The above result provides a sufficient condition for the absence of multiple equilibria of a type of SSI modules. But this condition cannot be satisfied by all such SSI modules. In other words, some SSI modules can actually admit multiple equilibria. We will give such an example. The SSI module is shown in Figure 4. Let *C*_0_, *C*_1_, *C*_2_ and *C*_3_ represent the concentration of the metabolites *A*, *B*, *C* and *D*, *C* = (*C*_1_, *C*_2_, *C*_3_)*^τ^*.

Then we can get the model,

*dC*_1_/*dt* = *v*_1_ – *v*_2_ – *v*_3_ (13a)

*dC*_2_/*dt* = *v*_2_ – *v*_4_ (13b)

*dC*_3_/*dt* = *v*_3_ – *v*_5_, (13c)

where

Denote

Then the Jacobian matrix of the rate function *R*(*C*;*P*) in (13) can be write as

Consequently,

Note that that  ,  ,  and  are positive,  and  are negative. Then,  can be zero for some *C* and *P*, which implies the system (13) may have multiple equilibria. In fact, for the parameter values listed in Table 1, the system has three equilibria,

*C*_1_ = 0.35000 *C*_2_ = 0.72835 *C*_3_ = 2.44647, (14)

*C*_1_ = 0.35438 *C*_2_ = 2.48512 *C*_3_ = 0.78621, (15)

*C*_1_ = 0.34243 *C*_2_ = 1.11200 *C*_3_ = 1.34743. (16)

**Table 1:** Parameter values

parameter	value	parameter	value	parameter	value
*V_max_*_1_	2.6	*K*_1_	0.14	*n*_1_	1
*V_max_*_2_	3.7	*K*_2_	0.23	*n*_2_	2
*V_max_*_3_	4.3	*K*_3_	0.23	*n*_3_	2
*V_max_*_4_	1.3	*K*_4_	0.29	*n*_4_	1
*V_max_*_5_	1.5	*K*_5_	0.27	*n*_5_	1
*K_C_*_2_	5.4	*K_u_*_2_	9.8	*C*_0_	1
*K_C_*_3_	6.2	*K_u_*_3_	8.7		

The parameter values used in the simulation of the numeric example.

The Jacobian matrix of the rate function *R*(*C*; *P*) at the equilibrium (14) is(17)

Its eigenvalues are

*λ*_1_ = –9.1666, *λ*_2_ = –0.1730, *λ*_3_ = –0.0267.

They are all negative numbers, which implies that the system (13) is asymptotically stable at the equilibrium (14).

The Jacobian matrix of the rate function *R*(*C*;*P*) at the equilibrium (15) is(18)

And its eigenvalues are

*λ*_1_ = –8.8773, *λ*_2_ = –0.3005, *λ*_3_ = 0.0209.

**Figure 5:**
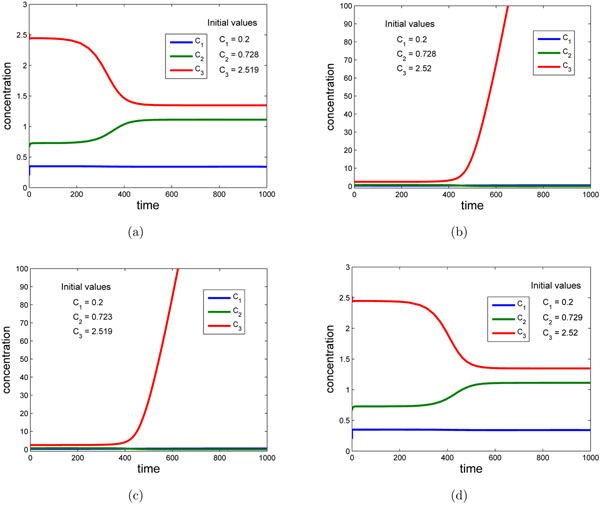
**Simulation of the example** The dynamic behaviors of the system (13) starting from four different initial values. The model parameters are listed in Table 1. The initial values of each figure are, respectively: (a) *C*_1_ = 0.2, *C*_2_ = 0.728, *C*_3_ = 2.519. (b) *C*_1_ = 0.2, *C*_2_ = 0.728, *C*_3_ = 2.520. (c) *C*_1_ = 0.2, *C*_2_ = 0.723, *C*_3_ = 2.519. (d) *C*_1_ = 0.2, *C*_2_ = 0.729, *C*_3_ = 2.520.

The first two are negative and the last one is positive. This means the system (13) is not stable at the equilibrium (15). Figure 5 shows its dynamic behaviors starting from four different initial values around the equilibrium (15). Figure 5(a) shows that the trajectory is converged when it starts from the initial values *C*_1_ = 0.2, *C*_2_ = 0.728 and *C*_3_ = 2.519. If we take a small change on the initial value for *D*, i.e. take *C*_1_ = 0.2, *C*_2_ = 0.728 and *C*_3_ = 2.520, then the trajectory would be diverged, see Figure 5(b). And then the trajectory will be converged again if we take a small change on the initial value for *C*, i.e. take *C*_1_ = 0.2, *C*_2_ = 0.729 and *C*_3_ = 2.520, see Figure 5(d)

## Conclusions

The multi-equilibrium property of metabolic networks is of great practical significance and difficult to be investigated biologically or theoretically. To study it, we proposed a structure-oriented modularization framework: viewing a metabolic network as an assembly of basic building blocks with particular structures, and investigating the multi-equilibrium property of the original network by studying the characteristics of the basic modules and their interactions. The SSI module is one of the four types of basic building blocks, whose multi-equilibrium property was studied in this paper.

Due to the complexity of its topological structure, the strong coupling between each metabolite and the intricacy of the reaction mechanism, it is a difficult task to analyze the dynamic properties of SSI modules. In particular, comparing with SSN modules, there exists negative feedbacks in SSI modules caused by inhibitions, which makes the module structure and the reaction mechanism much more complicated. This paper mainly discussed one common type of SSI modules in which each reaction has no more than one inhibitor, which is considered as the first step towards elucidating the design principle of metabolic networks in living organisms. In the near future, we will further discuss the SSI modules in which there are reactions with more than one inhibitor. In addition, the main idea of this work can be extended to study the problem of networkomics (or netomics) which covers all stable forms of biomolecular networks [1,2] not only at different biological conditions but also at different spatiotemporal situations.

## Methods

### Proof of Lemma 2

*Proof*. Note that the Jacobian matrix  . Then(19)

Now, we will prove (i). Assume, to arrive a contradiction, that *F* was not injective on ***D*** for some  , which means that there would exist  such that  . The conditions in (i) implies that there exist  ,  and a nonzero vector  such that (10) holds. Combining with (19), we have

This implies that the Jacobian matrix  is singular, which contradicts the condition. Hence, *F*(*x*, *p*) is injective on ***D*** for all *p* ∈ ***P***.

(ii) can be proved similarly.

### Proof of Lemma 3

*Proof*. The partial derivative of *f_k_*(*x*, *p*) with respect to *x_i_* is

where

Let  .Then(20)

where(21)

Denote(22)

Now we will first show the necessity. By Lemma 2(i), it to sufficient to show that for any  and  , there exist  ,  and a nonzero vector  such that the following equation holds for all *k* ∈ *N*,(23)

that is, (10) holds.

*Y_k_* contains the parameters  ,  ,  ,  and  while *k* ∈ *N*_2_, the parameters  and  while *k* ∈ *N*_3_, and the parameters  ,  ,  and  while *k* ∈ *N*_4_, and does not contain the parameter  . *Z_k_* contains the parameters  ,  ,  ,  and  while *k* ∈ *N*_2_, the parameters  and  while *k* ∈ *N*_3_, the parameters  ,  ,  and  while *k*∈*N*_4_, and does not contain the parameter  . For given  ,  ,  ,  ,  , and  , take  and  ,and define  and  as follows,

Then  implies  is a nonzero vector. If there exist  ,  and  such that *Y_k_* and *Z_k_* have the same sign, then  will satisfy (23) if *Y_k_* = *Z_k_* = 0; or (23) will hold by taking  if *Y_k_* ≠ 0. So it is sufficient to show there exist  ,  and  such that *Y_k_* and *Z_k_* have the same sign.

Case *k* ∈ *N*_1_*:* Equations (21) and (22) imply *Y_k_* = *Z_k_* = 0 always holds.

Case *k* ∈ *N*_2_: Now,  is decreasing with respect to *x_q_k__*. Thus, if  , then *Z_k_* < 0 and  , and consequently,  , then *Z_k_* >*0* and  , and consequently, *Y_k_* > 0; if  , then *Z_k_* = *0* and  , and consequently, *Y_k_* = 0.This implies that *Y_k_* and *Z_k_* have the same sign.

Case *k* ∈ *N*_3_: Note that  is increasing with respect to *x_r_k__*. Similar to the proof of the case *k* ∈ *N*_2_ we can show that *Y_k_* and *Z_k_* have the same sign.

Case *k* ∈ *N*_4_*:* We will discuss this case according to the relationship between  and  , and that between  and .

If  and  , then  and  , and consequently,  . Noticing that  is increasing with respect to *x_r_k__* and decreasing with respect to *x_q_k__*, we have *Z_k_* <*0*. Hence, *Y_k_* and *Z_k_* have the same sign. The proof for the cases  and  and  and  is similar.

If  and  , then *Z_k_* > 0,  and  , and consequently, *Y_k_* > 0 for all. Hence, *Y_k_* and *Z_k_* have the same sign. The proof for  and  is similar.

If  and  , then  and  . Denote

where

Since *W_k_* > 0, it is sufficient to find some  such that *η_k_* and *Z_k_* have the same sign. In fact, when  is sufficiently small and  is sufficiently large, we have *η_k_* > 0; and when  is sufficiently large and  is sufficiently small, we have *η_k_* < 0. Hence, there exist  ,  and  such that *η_k_* and *Z_k_* have the same sign, regardless of *Z_k_* > 0 or *Z_k_* < 0. The proof for  and  is similar.

Next, we will show the sufficiency. By Lemma 2 (ii) and the discussion in the proof of the necessity, it is sufficient to show for any  ,  and nonzero vector  , there exist  and  such that *Y_k_* and *Z_k_* have the same sign for all *k* ∈ *N*.

Take  and  . Define  and  as follows,

Then  is a nonzero vector implies  . Hence, it is sufficient to find some  such that *Y_k_* and *Z_k_* have the same sign for .

Case *k* ∈ *N*_1_*:* We have shown *Y_k_* and *Z_k_* are zero for all  and .

Case *k* ∈ *N*_2_ ∪ *N*_3_: Similar to the proof of necessity for the cases *k* ∪ *N*_2_ and *k* ∪ *N*_3_, respectively, we can show that *Y_k_* and *Z_k_* have the same sign.

Case *k* ∪ *N*_4_*:* and  imply  ,  and  , which indicate *Z_k_* < 0 has the same sign with *Y_k_*. Similarly, one can show that *Y_k_*and *Z_k_* have the same sign for the cases  and  ,  and  ,  and .

When  and  , or  and  , one can get that *Y_k_* and *Z_k_* have the same sign by noticing the monotonicity of  .  and  imply  and  . Noticing that  , we have  ,  and  . Thus, if *Y_k_* > 0, then we can find some  ,  and  such that

which implies *Z_k_* > 0. In contrast, if *Y_k_* < 0 we can also find some  ,  and  such that *Z_k_* < 0. Hence, *Y_k_* and *Z_k_* have the same sign. It is similar to show that for  and  .

### Proof of Theorem 1

*Proof*. Divide the reactions in *ℛ* into four classes,

*N*_1_ = {*j* : *j* ∈ {1,⋯, *m*}, there is no inhibitor of *A_j_* → *B_j_* ∈ *ℛ*, and *A_j_* is an input node}

*N*_2_ = {*j* : *j* ∈ {1,⋯, *m*}, there exists inhibitor of *A_j_* → *B_j_* ∈ *ℛ*, and *A_j_* is an input node}

*N*_3_ = {*j* : *j* ∈ {1,⋯, *m*}, there is no inhibitor of *A_j_* → *B_j_* ∈ *ℛ*, and *A_j_* is a state node}

*N*_4_ = {*j* : *j* ∈ {1,⋯, *m*}, there exists inhibitor of *A_j_* → *B_j_* ∈ *ℛ*, and *A_j_* is a state node}.

When each reaction in *ℛ* has no more than one inhibitor, the rate equations *v_j_*, *j* ∈ *N_k_*, *k* = 1, 2, 3, 4, confirms with the function *f_k_* in (12), respectively. Thus, the model (8) of this SSI module is a special case of the system (11), which means the results in Lemma 3 are still valid for such an SSI module.

## Authors contributions

HBL, JFZ and LC proposed the main idea. HBL developed theoretical results and drafted the manuscript. JFZ and LC gave valuable suggestions. All authors wrote and approved the manuscript.

## Competing interests

The authors declare that they have no competing interests.
